# Therapeutic Plasmapheresis: A Treatment Modality in Severe Hypertriglyceridemia in Adolescence

**DOI:** 10.7759/cureus.17341

**Published:** 2021-08-20

**Authors:** Nayab Afzal, Shamim Kausar

**Affiliations:** 1 Chemical Pathology, National Medical Centre, Karachi, PAK; 2 Intensive Care Unit, National Medical Centre, Karachi, PAK

**Keywords:** severe hypertriglyceridemia, therapeutic plasmapheresis, acute pancreatitis, diabetic ketoacidosis (dka), metabolic syndrome

## Abstract

Severe hypertriglyceridemia (SHTG) is defined as plasma triglycerides >1000 mg/dl. It is a rare and understudied condition in children and adolescents. SHTG in pediatric patients may occur as a result of certain genetic disorders of enzymes involved in triglyceride synthesis and metabolism, or it may be seen secondary to uncontrolled diabetes, obesity, metabolic syndrome, or certain medications. SHTG can cause recurrent abdominal pain and acute pancreatitis. Rapid lowering of triglycerides is needed in SHTG to prevent these complications. However, there are no specific guidelines for the treatment of severe hypertriglyceridemia in children and adolescents. Here we report a case of a 16-year-old boy, who was diagnosed with metabolic syndrome three years back. He had a family history of diabetes type 2 and dyslipidemia. In the current case presentation, the patient presented with SHTG-induced acute pancreatitis, who was successfully treated using plasmapheresis. To our knowledge, no such case has ever been reported in Pakistan. Our case findings highlight the use of a less conventional method for the treatment of hypertriglyceridemia in children.

## Introduction

Triglycerides (TGs) are fatty acid esters of glycerol with several important physiological functions. Excessive amounts of TGs in the blood lead to hypertriglyceridemia (HTG). TG levels of 1000-1999 mg/dl are classified as severe hypertriglyceridemia (SHTG) [1]. It may cause xanthomas, hypertension, insulin resistance, or recurrent pancreatitis. Acute pancreatitis (AP) resulting from SHTG can lead to significant mortality and morbidity. It is crucial to identify SHTG as the cause of acute pancreatitis so that prompt treatment in this regard can be initiated [2]. Conventional management of severe HTG along with acute pancreatitis includes the use of hypolipidemic drugs and insulin/dextrose infusions [3]. However, these treatment options are slow to deliver results. Here we describe the successful use of therapeutic plasmapheresis to treat a case of SHTG complicated with acute pancreatitis in an adolescent child.

## Case presentation

We present a case of a 16-year-old adolescent male diagnosed with metabolic syndrome three years back. At the time of diagnosis, his BMI was 31.31 kg/m2, glycosylated hemoglobin (HbA1C) was 10.0%, fasting plasma glucose was 195 mg/dl, he was hypertensive, and his triglycerides were 450 mg/dl. He had a family history of dyslipidemia and type 2 diabetes at a young age. He was managed with dietary and lifestyle modifications, along with insulin therapy and fenofibrate. In the last seven months, he suffered from two episodes of acute pancreatitis. The specific cause of previous episodes of acute pancreatitis could not be ascertained as the patient was treated in another city. Both episodes of acute pancreatitis were managed with supportive treatment without life-threatening complications. This time he presented to the emergency room with severe abdominal pain, nausea, and vomiting for three days. Upon investigation, laboratory results showed metabolic acidosis, HbA1c 9.3% (<5.6%), triglyceride 1890 mg/dl (<150), cholesterol 206 mg/dl (<200), lipase 2519 U/L (13-60), amylase 739 U/L (<100), and lactate 5.2 mg/dl (0.5-2.2). CT scan abdomen showed mild ascites with fluid around the liver and spleen, with no evidence of intrahepatic or extrahepatic biliary duct dilatation. The pancreas appeared swollen with stranding of peripancreatic fat and there is increased attenuation of peripancreatic fat. A linear/cystic hypodense lesion was seen in the region of the tail of the pancreas measuring 1.0 x 0.9 cm, and another cystic lesion was noted in the head of the pancreas measuring 1.1 x 1.0 cm. These findings were consistent with recurrent pancreatitis. Urinalysis was positive for glucose and ketones. His abdominal imaging ruled out any biliary stone or cyst. Other common causes of acute pancreatitis like alcoholism and drugs were excluded through history. A diagnosis of diabetic ketoacidosis (DKA) along with triglyceride-induced acute pancreatitis was made. He was managed with intravenous insulin infusion (Humulin R 0.1 units/kg/hr). He was administered 120 units of regular insulin and 9 liters of positive fluid balance was maintained with intravenous hydration (0.9% normal saline) on the first day of admission, but his pain worsened and he rapidly deteriorated to have multiorgan failure including redistributive shock (blood pressure [BP] 90/60 mmHg and heart rate [HR] 150 beats/min), severe metabolic acidosis, disseminated intravascular coagulation, acute kidney injury, and hypocalcemia (Table 1).

**Table 1:** Various biochemical parameters during the course of admission * After the first session of therapeutic plasmapheresis. ** After the second session of therapeutic plasmapheresis. PCO2, partial pressure of carbon dioxide; PO2, partial pressure of oxygen; HCO3, bicarbonate; APTT, activated partial thromboplastin time; PT, prothrombin time; CRP, C-reactive protein.

Analytes	Day 1	Day 2	Day 3*	Day 4**	Day 5	Reference range
Random glucose (mg/dl)	652	339	259	191	207	70-140
Urea (mg/dl)	16	26	41	32	30	10-50
Creatinine (mg/dl)	0.8	1.5	1.3	1.1	1.0	0.5-1.2
Ph	7.41	7.11	7.32	7.41	7.43	7.35-7.45
PCO2 (mmHg)	32.9	26.1	25.4	29.8	31.9	35-46
PO2 (mmHg)	107	90	114	114	130	85-105
HCO3 (mmHg)	21.0	12.0	13.3	19.4	22.7	25-35
O2 saturation %	98.3	95.1	98.4	98.6	99.2	95-98
PT (sec)	15.4	24.7	26.0	13.1	11.2	Control = 10
APTT (sec)	22.0	35.6	70.0	41.0	32.4	Control = 27
Lactate (mg/dl)	5.2	6.0	1.99	1.48	1.3	0.5-2.2
CRP (mg/l)	188	345	234	108	92	<5
Calcium (mg/dl)	-	6.2	7.1	8.0	8.1	8.6-10.2
Magnesium (mg/dl)	-	1.5	1.9	2.0	1.9	1.7-2.7
Phosphorus (mg/dl)	-	1.5	2.4	4.6	5.0	2.5-5.0

**Figure 1:**
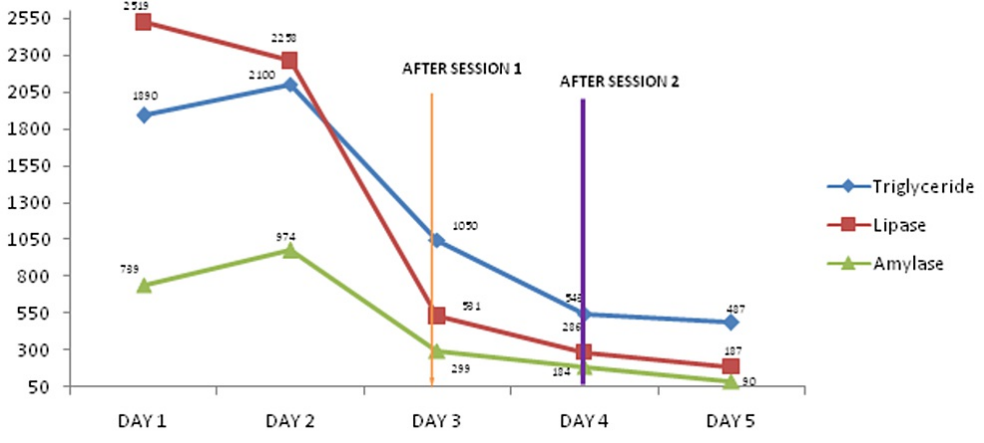
Triglyceride, lipase, and amylase levels before and after two sessions of plasmapheresis

The inflammatory markers and serum amylase and lipase levels worsened despite aggressive supportive treatment to failing organs, for which two sessions of plasmapheresis were done to treat severe hypertriglyceridemia as a cause of acute pancreatitis. The triglyceride level before plasmapheresis was 2100 mg/dl, which became 487 mg/dl on the day following only two sessions of plasmapheresis. His inflammatory markers and amylase and lipase levels declined rapidly after the first session of plasmapheresis (Figure 1).

Metabolic acidosis was treated with normal saline and 25% dextrose water. The patient gradually recovered from multiorgan failure with other supportive treatments. Genetic testing for familial hypertriglyceridemia is not available in Pakistan so could not be performed on our patient. The patient was discharged with the advice of maintaining an active lifestyle, dietary modifications, exercise, and weight reduction. Fenofibrate, niacin, and omega 3 fatty acids were prescribed to control the triglyceride levels in the body.

## Discussion

The complex and potentially life-threatening triad of DKA, SHTG, and acute pancreatitis (AP) has been previously reported, but the exact mechanism is not clearly understood. Debate is still going on about whether DKA is the cause or complication of AP [4]. Insulin deficiency leads to disorders of lipid and glucose metabolism and decreases the amount of lipoprotein lipase, an enzyme responsible for the hydrolysis of triglycerides. During DKA there is increased fat mobilization and reduced breakdown of triglycerides leading to hypertriglyceridemia [5-6]. Pancreatic lipase converts these triglycerides into free fatty acids. Normally these free fatty acids are nontoxic to the pancreas because they bind to albumin. However, when TG levels are extremely high they become cytotoxic to pancreatic cells and lead to AP. Acute pancreatitis in DKA can be explained by elevated toxic free fatty acid that damages the pancreas. On the other hand, hypertriglyceridemia due to some other causes like alcoholism or hereditary diseases of triglyceride metabolism can result in acute pancreatitis, which may decompensate diabetes leading to DKA [7]. In both conditions, the patient can present with the triad of DKA, AP, and SHTG.

Our patient presented with metabolic acidosis, abdominal pain, and severe hypertriglyceridemia. There was strong evidence of insulin deficiency as shown by elevated blood glucose, low bicarbonate, elevated urine glucose, ketone, and protein. Serum ketones were not checked due to lack of availability in our laboratory. In this case, DKA led to SHTG, which resulted in AP. Given the fact that DKA was the cause of SHTG and AP, traditional treatment with intravenous insulin and proper hydration was started. With the given treatment, an improvement was expected in symptoms, triglycerides, and other parameters; however, symptoms worsened and the patient deteriorated on day two of admission; this prompted the need for plasmapheresis, which could rapidly decrease the triglyceride levels and prevent severe complications like pancreatic necrosis. While regular treatment may take weeks to lower down the TG, this alternative nonpharmacological technique can achieve rapid reduction of TGs even in a single session [8].

**Figure 2:**
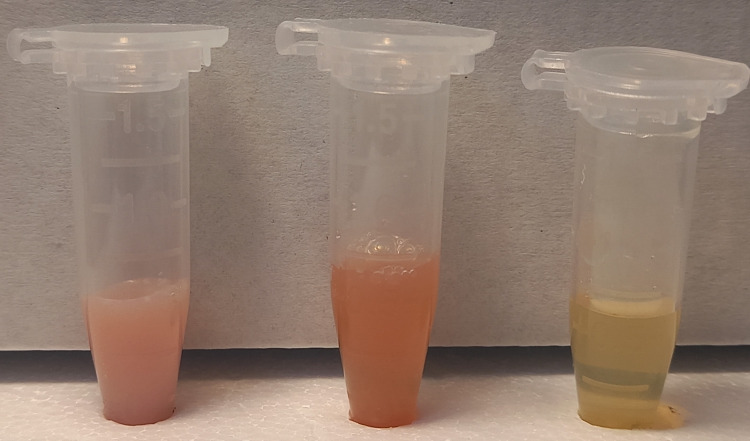
Physical appearance of serum samples collected before and after therapeutic plasmapheresis Starting from left: aliquot 1 is a sample before plasmapheresis, aliquot 2 is a sample after the first session of plasmapheresis, and aliquot 3 is a sample after the second session of plasmapheresis.

There is no clear guideline about the management of SHTG-induced AP in children. To our knowledge, no pediatric case with a triad of DKA, SHTG, and AP has been reported in Pakistan. However, a few internationally reported cases have shown promising results in children with IV hydration and insulin therapy [9-10]. In our patient, the sudden worsening of symptoms and risks of various life-threatening complications of SHTG (i.e., pancreatic necrosis, stroke, and ischemia) led to the initiation of plasmapheresis. Therapeutic plasmapheresis removes TG-rich lipoproteins from the plasma thus preventing worsening and relapse of AP [11]. The insulin infusion and IV hydration was continued and two sessions of therapeutic plasmapheresis were done on day three and day four. During each session of plasmapheresis, 3000 ml plasma by using formula (bodyweight x 70) x (1- hematocrit) was replaced, and six units of fresh frozen plasma (200 ml each) were used as replacement fluid. These early sessions resulted in fast resolution of symptoms, the decline in TGs, lipase, and amylase, and improvement in other biochemical parameters in the patient. No local or systemic adverse effects like a decrease in blood pressure, sweating, and abdominal cramps were noted. The massive decrease in TGs was also visible in the serum sample. Serum was cloudy white before plasmapheresis, it became less hazy after the first session and a clear sample was obtained after the second session of plasmapheresis (Figure 2).

## Conclusions

In our experience, early plasmapheresis proved to be beneficial for the adolescent boy, saving him from brutal complications of SHTG. Hence therapeutic plasmapheresis appears to be a useful option for rapid reduction of TGs in younger patients. However, the use of therapeutic plasmapheresis as monotherapy cannot be advocated as insulin infusion and hydration also play an important role in lowering triglyceride levels. Given that no large-scale studies are available, further trials are needed to determine the safety and effectiveness of therapeutic plasmapheresis in the treatment of SHTG in childhood and adolescence.
